# The growth of tumour xenografts in thymectomized high dose irradiated mice reconstituted with syngeneic bone marrow cells incubated with anti-thymocyte serum.

**DOI:** 10.1038/bjc.1976.12

**Published:** 1976-01

**Authors:** C. R. Franks, D. Bishop, D. Reeson


*Br. J. Cancer* (1976) **33**, 112

***Short Communication***

### THE GROWTH OF TUMOUR XENOGRAFTS IN THYMECTOMIZED HIGH DOSE IRRADIATED MICE RECONSTITUTED WITH SYNGENEIC BONE MARROW CELLS INCUBATED WITH ANTI-THYMOCYTE SERUM

C. R. FRANKS*, D. BISHOP AND D. REESON

*From the *ICRF Breast Cancer Unit, Guy's Hospital, London, SE1 9RT, and Viral Products, National Institute for Biological Standards and Control, Holly Hill, London, NW3 6RB*

Received 27 August 1975 Accepted 23 September 1975

It is well established that tumour xenografts, including tumours from patients with primary cancer, can be grown in thymectomized, irradiated mice reconstituted with syngeneic bone marrow cells (T-B+) (Castro, 1972; Franks, Perkins and Holmes, 1973; Cobb and Mitchley, 1974). In all these studies, a method similar to that of Miller *et al.* (1963) has been used to produce the T-B+ mice, although the technique proposed by Miller and his colleagues formed the basis for investigations unrelated to tumour growth. Nevertheless, a high degree of positive " takes " has been reported using this method. It has also been observed that some tumours regress immediately after implantation whilst the same tumours, in other T-B+ mice, continue to grow. The reasons for this variation in growth are not entirely clear. However, it is possible that this may be due in part to the fact that, following irradiation of the mice at 900 rad, some 5% of viable thymus-dependent (T) lymphocytes are known to survive (Raff and Wortis, 1970). A further 5% of T cells are introduced with the syngeneic bone marrow cell inoculum (Doenhoff and Davies, 1971), making a residual pool of some 10% T cells. Although the procedure used to produce the T-B+ mice is standardized as far as possible, clearly there is likely to be some variation between individual mice and heterogeneity which may account for the differential growths of the tumours observed. Perhaps of more significance is the 10% pool of circulating T cells. In order to try and eliminate the two possible sources contributing to the pool, some mice in the study were irradiated at 1200 rad and the remainder at the standard 900 rad. In addition, some mice from each group received bone marrow cells previously incubated with rabbit anti-mouse thymocyte serum (ATS); the rest received untreated bone marrow cells.

### MATERIALS AND METHODS

Female CBA mice from the specific pathogen-free colony at the National Institute for Medical Research, Mill Hill, London, NW7 4RD were used in the study. They were thymectomized at 4 weeks of age and whole body irradiated with a lethal dose (900 rad and 1200 rad) from a $^{60}Co$ source 2 weeks later. Some mice from each irradiation group were reconstituted with $10^7$ syngeneic bone marrow cells which had been previously incubated with 0·5 ml of ATS for 3 h at 37°C. The remaining mice received an inoculum of $10^7$ untreated syngeneic bone marrow cells. Sixty mice were used in the study.

The anti-thymocyte serum was prepared by the method of Levey and Medawar (1966). Its efficacy *in vivo* was tested by the method of Stanbridge and Perkins (1969). Two tumour lines were studied. The HeLa cells used were originally obtained from the Central Public Health Laboratories, Colindale, but were subsequently passaged in the laboratory. They were shown to be free from mycoplasma. Each mouse received 2 × $10^5$ cells in a 0·2 ml inoculum by the subcutaneous route. The remaining mice received pieces of solid tumour at least 9 $mm^3$ from a papillary cell carcinoma of the human bladder on its fourth passage, also by the subcutaneous route. Histology confirmed the presence of a highly anaplastic tumour.

Growth of both implanted tumours was assessed by measuring 2 axes at rightangles using vernier callipers. The product of the axes was designated the surface area of the tumour.

### RESULTS AND DISCUSSION

Figures 1 and 2 demonstrate the growth patterns observed from the implanted HeLa and bladder tumours respectively. Figure 1 shows that ATS treatment of the bone marrow cells used to reconsitute the mice irradiated at 900 rad enhanced the growth of HeLa tumours. This enhancement persisted for more than 50 days after implantation (+ 50). By Day + 63, the tumours implanted in the 900 rad mice showed sustained growth whereas the tumours in the 900 rad + ATS mice had regressed. A similar enhanced growth pattern was observed in the 1200 rad + ATS mice but by Day + 47 there was little difference in size between these tumours and those in the 1200 rad mice. Unfortunately, the 1200 rad + ATS mice died at Day + 50. By Day + 63, the 1200 rad mice showed a rapid growth rate, which was maintained to the end of the study. However, this was always below the growth observed in the 900 rad or 900 rad + ATS groups of mice.

In Fig. 2, enhanced growth of the implanted tumours was once again ob-

FIG. 1.—The subcutaneous growth of HeLa tumours in T-B+ mice irradiated at 900 rad and 1200 rad, half of which were reconstituted with bone marrow cells pretreated with ATS.

FIG. 2.—The subcutaneous growth of a papillary cell carcinoma of the human bladder in T-B+ mice irradiated at 900 rad and 1200 rad, half of which were reconstituted with bone marrow cells pretreated with ATS.

served in the 900 rad + ATS mice, when compared with the 900 rad mice. Furthermore, unlike the HeLa tumours, this enhancement of growth was maintained to the end of the study period at Day + 70, and became more pronounced with time. The tumours in the 1200 rad group of mice were initially slow to grow but by Day + 70 the growths observed were exceeded only by the 900 rad + ATS mice. In this study, the 1200 rad + ATS mice died within a few days of implantation of the tumour.

From the results, it is clear that the combination of irradiation and ATS incubated bone marrow cells enhances subcutaneous growth of tumour xenografts derived from HeLa cell suspensions and solid tumour implants. This suggests that the 5% T cell population introduced with the bone marrow cell inoculum may be the significant factor controlling the inhibition of implanted tumour growth. In this study, it is not clear why the HeLa tumours started to regress in the 900 rad + ATS mice at Day + 63. However, Medawar (1969) has shown that ATS treated mice do not recover full immunological competence until 50 days after the end of ATS treatment. It is therefore possible that the combination of a return to immunological competence and the relatively small tumour load present is responsible for the gradual regression observed. This did not occur in the bladder tumour at 900 rad + ATS because by Day + 63 the surface area of the tumours was 4 times as great as in the mice with the HeLa tumours, thus indicating a well established graft. In the 900 rad mice, growth was slow in both tumour groups. It is suggested this is due to the presence of the small pool of immunologically competent cells introduced with the bone marrow cell inoculum, against which the establishment of tumour growth is achieved.

The comparatively slow growth rates initially obtained at 1200 rad (with or without ATS) may be due to subcutaneous damage following irradiation, thus inhibiting nutrition of the implanted tumours. Even after a recovery period, high irradiation levels do not appear to be superior to the lower levels of irradiation used. The failure to produce surviving 1200 rad + ATS mice in the solid tumour groups is thought to be due to the combined effect of 1200 rad, ATS, and ether anaesthesia. Ether anaesthesia was not required when HeLa cells were injected. In this particular study, there was no observed variation in the inter-mouse survival of tumours.

The combination of 900 rad + bone marrow cells incubated with ATS enhanced growth of implanted tumours and, in the case of solid tumours, sustained enhanced growth in excess of 22% at Day + 70 when compared with the 900 rad mice. The use of bone marrow incubated with ATS (rather than bone marrow alone) is therefore considered to be a worthwhile addition to the standard technique for growing tumour xenografts.

### REFERENCES

Castro, J. E. (1972) Human Tumours Grown in Mice. *Nature, New Biol.*, **239**, 83.

Cobb, L. M. & Mitchley, B. C. V. (1974) Growth of Human Tumours in Immune Deprived Mice. *Eur. J. Cancer*, **10**, 473.

Doenhoff, J. J. & Davies, A. J. (1971) Reconstitution of the T-cell Pool after Irradiation of Mice. *Cell Immunol.*, **2**, 82.

Franks, C. R., Perkins, F. T. & Holmes, J. Thornton (1973) Subcutaneous Growth of Human Tumours in Mice. *Nature, Lond.*, **243**, 91.

Levey, R. H. & Medawar, P. B. (1966) Nature and Mode of Action of Antilymphocyte Serum. *Proc. natn. Acad. Sci. U.S.A.*, **56**, 1130.

Medawar, P. B. (1969) Antilymphocyte Serum. Its Properties and Potential. *Hosp. Pract.*, **4**, 26.

Miller, J. F. A. P., Doak, M. A. & Cross, A. M. (1963) Role of the Thymus in Recovery of the Immune Mechanism in the Irradiated Adult Mouse. *Proc. Soc. exp. Biol. Med.*, **112**, 785.

Raff, M. C. & Wortis, H. H. (1970) Thymus Dependence of the Theta Bearing Cells in the Peripheral Lymphoid Tissues of Mice. *Immunology*, **18**, 931.

Stanbridge, E. J. & Perkins, F. T. (1969) Tumour Nodule Formation as an *in vivo* Measure of the Suppression of the Cellular Immune Response by ALS. *Nature, Lond.*, **221**, 80.


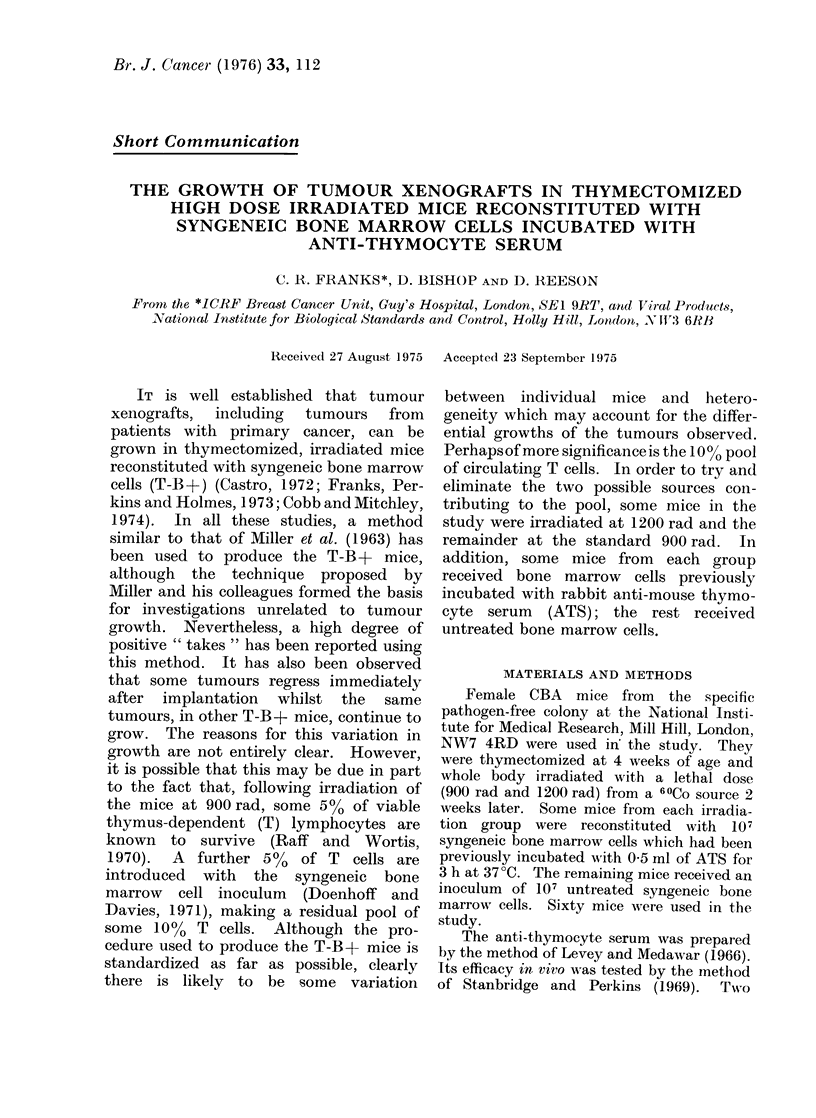

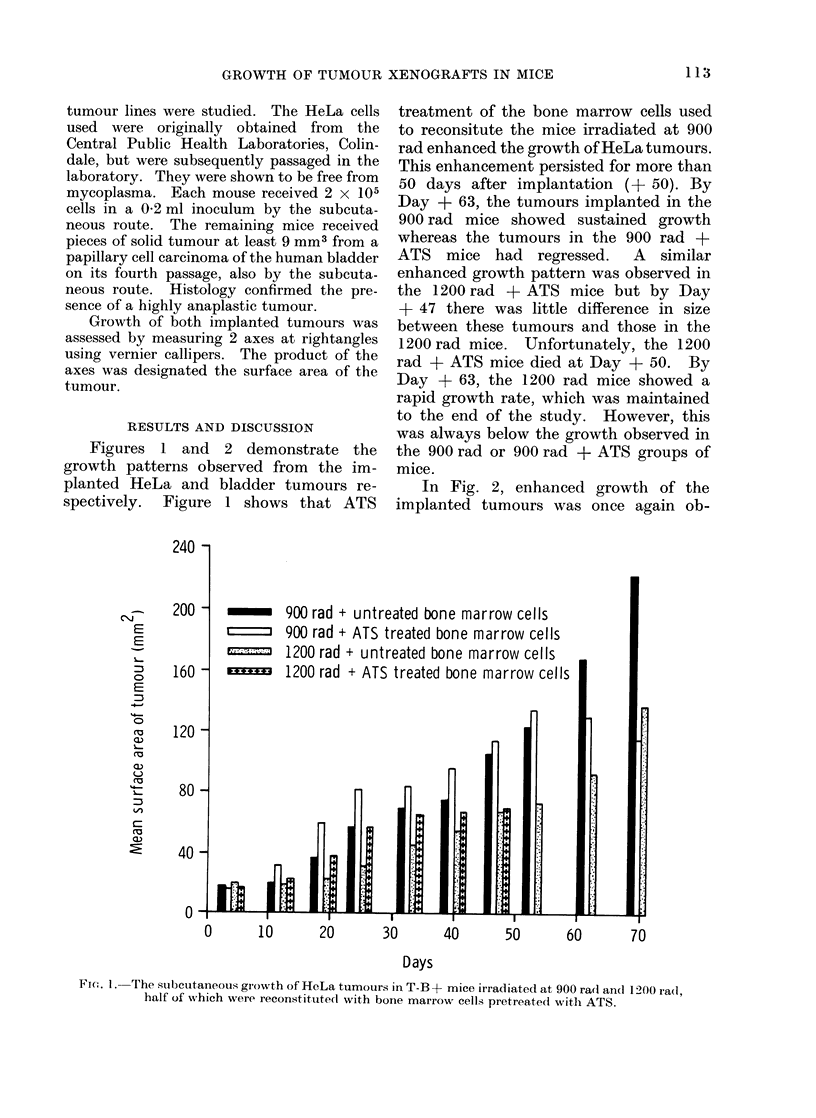

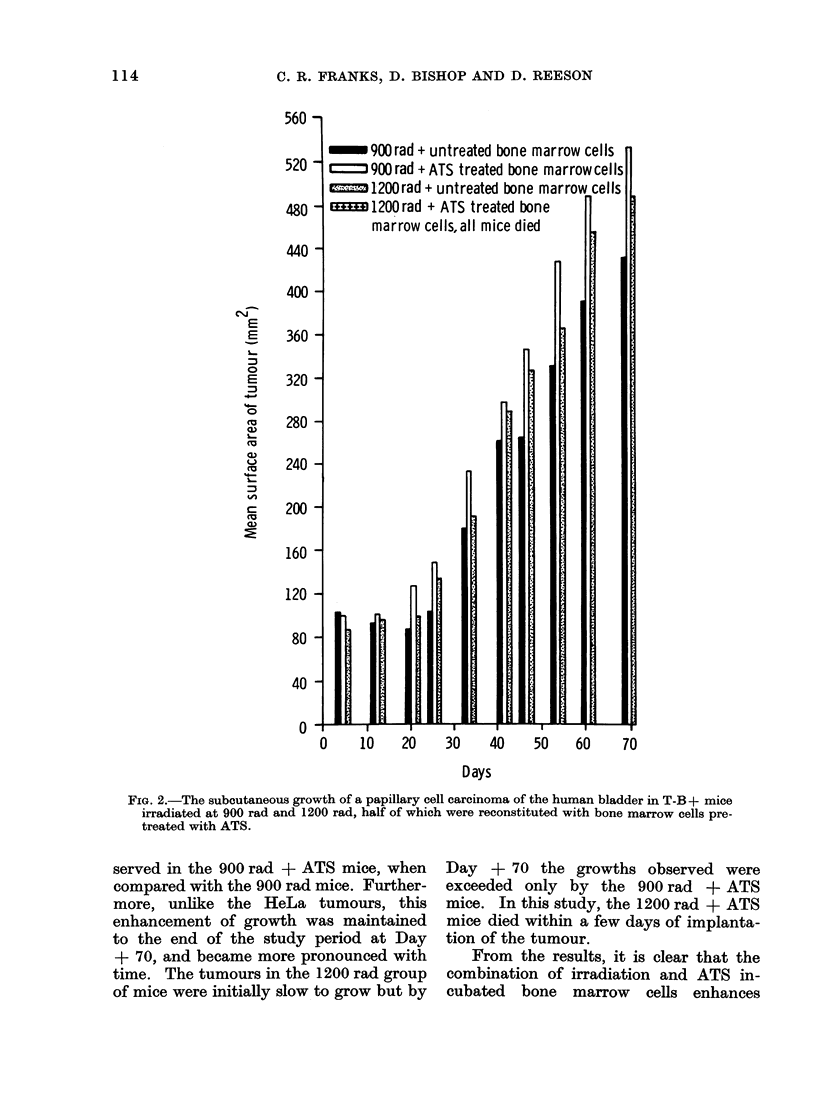

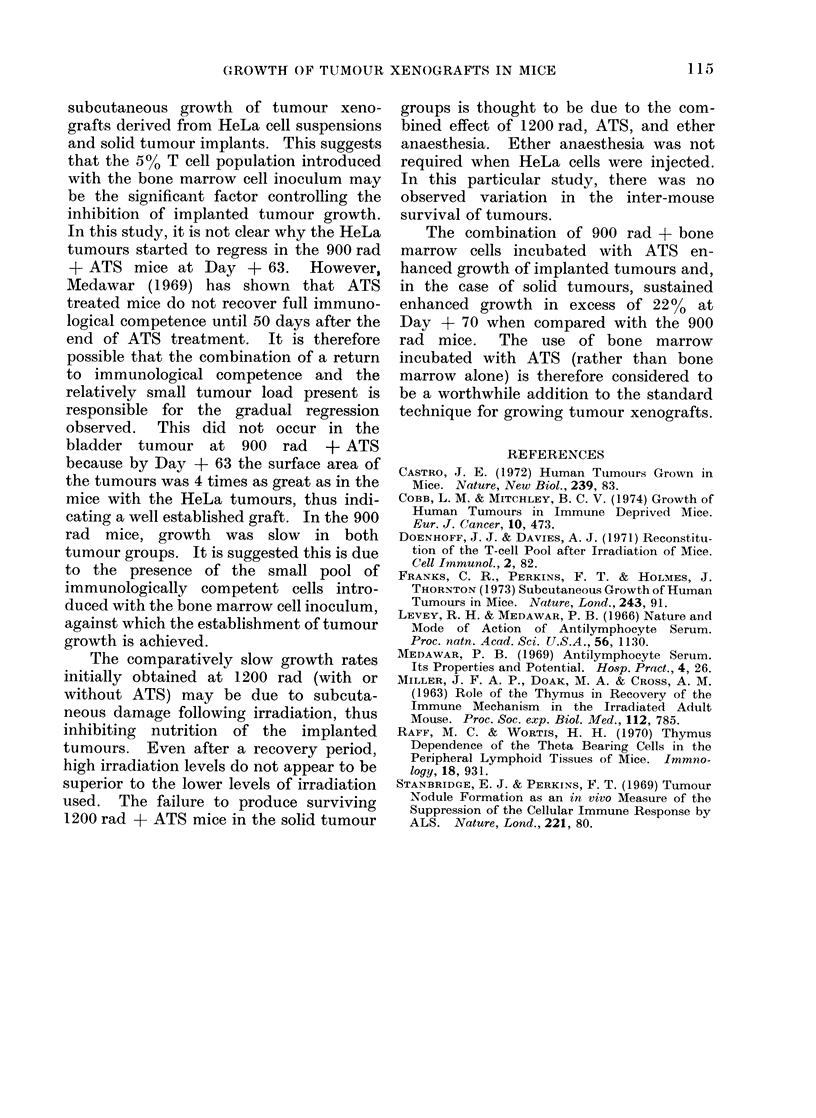


## References

[OCR_00375] Castro J. E. (1972). Human tumours grown in mice.. Nat New Biol.

[OCR_00379] Cobb L. M., Mitchley B. C. (1974). The growth of human tumours in immune deprived mice.. Eur J Cancer.

[OCR_00384] Doenhoff M. J., Davies A. J. (1971). Reconstitution of the T-cell pool after irradiation of mice.. Cell Immunol.

[OCR_00394] Levey R. H., Medawar P. B. (1966). Nature and mode of action of antilymphocytic antiserum.. Proc Natl Acad Sci U S A.

[OCR_00408] Raff M. C., Wortis H. H. (1970). Thymus dependence of theta-bearing cells in the peripheral lymphoid tissues of mice.. Immunology.

[OCR_00414] Stanbridge E. J., Perkins F. T. (1969). Tumour nodule formation as an in vivo measure of the suppression of cellular immune response by antilymphocytic serum.. Nature.

